# Perceptions of Saudis Toward Participating in the COVID-19 Convalescent Plasma Clinical Trial

**DOI:** 10.7759/cureus.48879

**Published:** 2023-11-16

**Authors:** Mariam M Al Eissa, Fahad Almsned, Raghad AlQurashi, Safaa M Alsanosi, Asim M Alshanberi, Nada Saleh, Ahdab A Alsaieedi, Reem R Alkharji, Amr J Halawani

**Affiliations:** 1 Medical School, Alfaisal University, Riyadh, SAU; 2 Molecular Genetics, Public Health Authority, Public health Lab, Riyadh, SAU; 3 Epidemiology and Public Health, King Fahad Specialist Hospital, Research Centre, Dammam, SAU; 4 Research and Development, NovoGenomics, Riyadh, SAU; 5 Molecular Genetics, Public Health Lab, Public Health Authority, Riyadh, SAU; 6 Pharmacology and Toxicology, Faculty of Medicine, Umm Al-Qura University, Makkah, SAU; 7 Cardiovascular and Metabolic Health, University of Glasgow, BHF Glasgow Cardiovascular Research Centre, Glasgow, GBR; 8 Community Medicine, Faculty of Medicine, Umm Alqura University, Makkah, SAU; 9 General Medicine Practice, Batterjee Medical College, Jeddah, SAU; 10 College of Medicine, King Saud Bin Abdulaziz University for Health Sciences, Riyadh, SAU; 11 King Abdullah International Medical Research Centre, Ministry of National Guard Health Affairs, Riyadh, SAU; 12 Medical Laboratory Sciences, Faculty of Applied Medical Sciences, King Abdulaziz University, Jeddah, SAU; 13 Vaccines and Immunotherapy Unit, King Fahd Medical Research Centre, King Abdulaziz University, Jeddah, SAU; 14 Research, Princess Nourah Bint Abdulrahman University Health Sciences, Research Centre, Riyadh, SAU; 15 Laboratory Medicine, Faculty of Applied Medical Sciences, Umm Al-Qura University, Makkah, SAU

**Keywords:** convalescent plasma, saudi population, clinical trial, plasma, covid-19

## Abstract

Introduction

The COVID-19 pandemic has been a major public health and economic issue worldwide. Even though vaccines have been developed to reduce the spread of the infection, treating patients remains a significant challenge. This study aims to measure the perceptions of Saudis toward participating in the COVID-19 Convalescent Plasma Clinical Trial.

Method

A cross-sectional study measuring the perceptions of Saudis toward participating in the COVID-19 Convalescent Plasma Clinical Trial was conducted with participants who had recovered from COVID-19. The study used an online questionnaire covering variables related to demographics, awareness, attitudes, perceptions, and plans for improvement.

Results

A sample of 1,051 participants participated in the questionnaire. A total of 85% had recovered from COVID-19, only 2.76% had participated in clinical trials before, and 83.44% would participate if they were advised or knew about them. The participants showed a high level of education, with 88% having obtained a degree and most used social media. The results can be biased toward the participants who get their knowledge from social media and hope to learn about things on social media. The gap in knowledge about clinical trials among the participants indicated that certain age groups could be targeted through channels where they communicate the most.

Conclusion

Engaging the community in clinical trials and educating others about their value by sharing experiences would help promote clinical trials and activate donations.

## Introduction

The COVID-19 outbreak caused by the newly emerged strain of coronavirus, severe acute respiratory syndrome-coronavirus-2 (SARS-CoV-2), has been a rapidly evolving situation worldwide, a health concern that threatens the lives of thousands of people and a major economic issue [1]. Respiratory symptoms are the major complication causing the morbidity rate of around 604,190,052 cases and 6,494,877 deaths worldwide [2,3]. Usually, an epidemic has considerable effects, and this pandemic is causing burdens on healthcare, the economy, and governments. Moreover, the impact extends to the public, where psychological distress causes anxiety, post-traumatic stress disorder, and depression [4].

Although vaccines have been developed to reduce the spread of the infection, treating patients remains a significant challenge. Guidelines for different treatment strategies for COVID-19 have been released during the pandemic, including antiviral, hydroxychloroquine, steroid, anticoagulant, and other supportive therapies [5]. However, most recommended treatments have not been proven effective in current large-scale trials [6].

Convalescent plasma (CP) is one of the investigational treatments that has been used for severe cases of COVID-19. CP is collected from a patient who has recovered from COVID-19 and usually contains immune-modulatory cytokines, neutralizing antibodies, and autoantibodies against SARS-CoV-2 [7-10]. This therapeutic strategy is based on injecting plasma from recovered subjects into severely affected patients to reduce the inflammatory process associated with COVID-19 infection and reduce the infection progress [10].

The concept of CP therapy is not novel; it has a long history and a strong biological basis (Casadevall & Pirofski, 2020). CP has been used in small numbers of patients during recent viral epidemics, such as the 2003 SARS epidemic, the 2009 “swine flu” epidemic, and the 2012 Middle East Respiratory Syndrome (MERS) outbreak [11-14]. In the 2009 influenza A virus pandemic, the infection was associated with a lower mortality rate in those who received CP therapy [15]. Moreover, several Ebola patients were treated with CP during the outbreak in 2014-2015 [16].

Immune responses to various viral antigens are observed in patients who have recovered from viral infection and have developed polyclonal antibodies. Some of these polyclonal antibodies will probably neutralize the virus and prevent it from spreading [17]. Antibody-based therapies against COVID-19 are now among the most extensively studied and used worldwide, and several clinical trials have been conducted to evaluate the efficacy and safety of CP [18]. According to Saudi Arabia's Vision 2030, reducing COVID-19 morbidity and mortality cost-effectively will lead to enhanced healthcare and self-sufficiency in treating major diseases that afflict large populations [1]. CP is considered safe and possibly a feasible treatment option, especially in emergencies where treatment is still unavailable, as seen with the COVID-19 outbreak [16,17]. However, further studies are needed to investigate the efficacy and safety of CP. This study aims to measure the perceptions of Saudis toward participating in the COVID-19 Convalescent Plasma Clinical Trial.

## Materials and methods

Ethical approval

This study was conducted following the Declaration of Helsinki and approved by the Institutional Review Board at the Public Health Authority SCDC Riyadh, Saudi Arabia, with approval number IRB-A02-2020 (202011171).

Study design

A cross-sectional study was conducted among patients infected with COVID-19 in Saudi Arabia in 2021. They were randomly approached by sending them an electronic questionnaire. The purpose of the research was explained to participants in the questionnaire. Participants electronically signed a consent form before filling out the survey.

Questionnaire tool

An online survey titled Plasma project - Google Forms was developed for the current study to assess four main domains: the study sample’s demographics, including gender, age group, nationality, education level, province of residence, income, healthcare sector employment/background, and previous participation in clinical trials; the general population's perceptions toward participation in a clinical trial, which included eight questions regarding previous COVID-19 infection experience, recovery, previous plasma donation, the national clinical trial awareness level, and participation wellness (S1); the awareness level toward participation in a clinical trial, including motivational and psychological aspects (S2:A and S2:B); and suggestions to promote the future awareness toward participation in a clinical trial. The questionnaire was designed in English and then translated into Arabic, the local language, by proficient speakers of both languages and reviewed to suit the general population.

Study populations

Inclusion

Patients infected with COVID-19 in Saudi Arabia in late 2020. Symptomatic patients over 18 years of age were selected, and participants included Saudis infected with COVID-19 outside Saudi Arabia.

Exclusion

Asymptomatic patients and those under 18 years old were excluded.

Sample size and data collection

We used the n4Studies [19] online tool to calculate the proper sample size (n=385 participants). Data are treated anonymously by avoiding collecting any personal identification and disclosing the data. All data in this project was saved in secured electronic files, shared with the Saudi Public Health Authority, and discarded at the study's end. Only the research team’s principal investigators can access collected data and information relevant to the study. Data were collected between late October 2020 and late January 2021 via a Google form. The survey was randomly distributed as an online version in Arabic for those who confirmed COVID-19 infection. The online survey was promoted through WhatsApp and Twitter. The survey was directed at the general Saudi Arabian population and could be completed anonymously. To avoid multi-submissions, one survey per IP address was allowed.

Statistical analyses

Given how little research has been examined, the analyses were mostly descriptive. The data were reviewed and statistically analyzed using R coding language to calculate a number (N), percentages (describe categorical data), mean, median, and standard deviation (SD). Where appropriate, non-parametric Chi-square tests were performed to assess the categorical responses. Plots were generated using the ggplot2 (v 3.3.3) and plotrix (v 3.8-1) R packages [20,21]. We used the dplyr (v 1.0.2) and tidyverse (v 1.3.0) R packages for data manipulation (to manipulate the data, R provides a library called dplyr and tidyverse which includes many built-in methods to manipulate the data) (22,23). A p-value of <0.01 was considered statistically significant.

## Results

**Figure 1:**
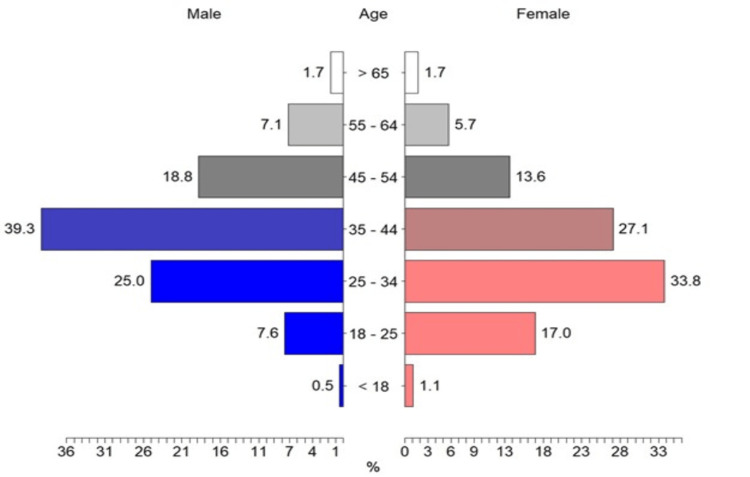
Age distribution comparing males and females

A total of 1,065 individuals completed the online survey. Fourteen participants were excluded from the study due to incomplete responses, leaving a total sample of 1,051 participants: 631 males (60.04%) aged 18 to 70 years (M = 35.75, SD = 10.96) and 420 females (39.96%) aged 18 to 66 years (M = 39.28, SD = 10.42). As shown in Figure 1, the greatest number of male participants are between 35 and 44 years old and the greatest number of females are between 25 and 34 years old. Saudis comprise 92.2% of the participants, whereas 7.8% are non-Saudis, as indicated in Table 1. The most frequent educational levels are 64.61% holders of a bachelor's degree and 23.5% holders of a Postgraduate graduate degree. Nearly all participants are from the Central, Eastern, Northern, Southern, and Western regions of Saudi Arabia, with rates of 48.81%, 14.65%, 9.51%, 7.9%, and 19.12%, respectively. Over half of the participants (52.62%) have high incomes of SAR 10,000 or more. One-third (34.35%) of the participants are healthcare workers, 41.77% have considerable healthcare knowledge overall, while the rest have no background. A total of 93.53% of the subjects have not previously participated in medical research.

**Table 1:** Sociodemographic data from 1051 participants

Category	Variable	N	%
Gender	Male	631	60.04
	Female	420	39.96
Age group (Years)	< 18	9	0.86
	18 – 24	139	13.23
	25 – 34	318	30.26
	35 – 44	336	31.97
	45 – 54	165	15.7
	55 – 65	66	6.28
	> 65	18	1.71
Nationality	Saudi	969	92.2
	Non-Saudi	82	7.8
Education	Illiterate	2	0.19
	Elementary	2	0.19
	Intermediate	7	0.67
	Secondary	114	10.85
	Bachelor	679	64.61
	Postgraduate	247	23.5
Province	Central	513	48.81
	Eastern	154	14.65
	Northern	100	9.51
	Southern	83	7.9
	Western	201	19.12
Income (SAR)	< 5,000	237	22.55
	5,000-9,999	261	24.83
	10,000-14,999	273	25.98
	> 15,000	280	26.64
Health sector employee	Yes	361	34.35
	No	690	65.65
Health sector background	Yes	439	41.77
	No	612	58.23
Participation in health research	Yes	68	6.47
	No	983	93.53

Recommendations to help improve participation in this clinical trial

**Figure 2:**
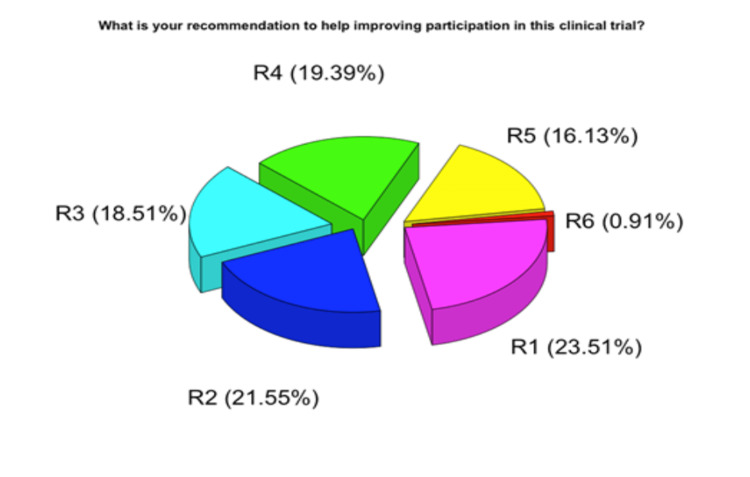
The pie chart shows the distribution of the participant’s answers to their recommendation for improving participation in plasma clinical trials. R1 = Improve social media channels, R2 = Sharing previous donor experience, R3 = Campaign, R4 = Video and infographics, R5 = Participation of schools and universities in education and awareness, R6 = Other

We addressed attitudes toward suggestions to improve participation in clinical trials. Many of the participants agreed that a focus on social media and advertising and sharing previous donor experiences, 23.51% and 21.55%, respectively, would improve participation, as shown on the pie chart in Figure 2. Some participants agreed on increasing campaigns, infographics/videos and enhancing education and awareness in schools and universities. The p-value shows a significant impact of 0.001 calculated by the Chi-square test, as indicated in Table 2.

**Table 2:** Statistical analysis of recommendations to help improve participation in this clinical trial

Variable	N	%	% of responses	% of cases	Chi-square test
R1 = Improve social media channels	771	23.51	23.5061	72.39437	c^2^ (5,N=1051) = 649.22, p<0.001
R2 = Sharing previous donor experience	707	21.55	21.55488	66.38498	
R 3 = Campaign	607	18.51	18.5061	56.99531	
R 4 = Video and infographics	636	19.39	19.39024	59.71831	
R 5 = Participation of schools and universities in education and awareness	529	16.13	16.12805	49.67136	
R 6 = Other	30	0.91	0.914634	2.816901	

Awareness level to participate in a CP clinical trial

The assessment was started by measuring the awareness level toward participation in a CP clinical trial by asking closed-ended or dichotomous questions (S1). The results of the survey showed that 83.54% of the participants had been diagnosed positive for COVID-19, including 14.64% who had not yet recovered and 17.6% who had not yet been free of symptoms for 14 days. Surprisingly, about 40% of the participants had never heard about plasma donation and only 4.28% of them had donated plasma. Although >50% of the participants were not aware of the national clinical trial of COVID-19 CP to treat critical cases, 47.19% had heard about it through healthcare providers (18.75%), friends enrolled in this study (4.23%), media (e.g., video, infographic and podcast) (36.49%), social media (32.66%) or through other sources (7.86%). A total of 83.44% of the participants would enrol in the clinical trial if their physician advised them to do so, and 2.76% would have enrolled even without receiving this survey.

Motivation to participate in a CP clinical trial

Aiming to understand the motivation behind participating in a CP clinical trial, we asked about reasons for participating in the clinical trial (Supplementary Table 3). The answers revealed that 90.49% think that CP can save patient lives and 67.55% would use the plasma product to treat relatives. Over four-fifths (81.54%) feel that this research raised emotional issues that they did not expect. Nearly all (94.77%) would participate for the positive impact of participating. Three-fifths (60.13%) of the participants worried about the experience of intense emotions during the research session, while 12.75% said they did not, and the rest replied “maybe’. Nearly three-quarters (72.42%) found participating in a clinical trial meaningful, while 5.04% did not, and the rest replied “maybe.” Almost the same percentage (72.50%) would join the clinical trial because it serves a good cause, while 6.66% would not. Finally, participating in clinical research was convenient for 38.06% and inconvenient for 20.65%.

Psychological aspects of participating in a CP clinical trial

We investigated the psychological reasons behind participating in a clinical trial by asking questions with “yes,” “no,” or “maybe” answers (Supplementary Table 4). Over one-third (38.63%) of the responses said that the researchers’ perspective influenced their decision to participate in the CP clinical trial, whereas up to 70% of participants chose the research views of the research team (yes=38.63%, no=18.65%, maybe=42.72%). Further questions asked about the participant being a critical case and needing help (yes=70.69%, no=11.70%, maybe=17.60%), engaging in national research (yes=80.40%, no=5.61%, maybe=13.99%), and believing the clinical research results will be useful to others (yes=76.02%, no=4.95%, maybe=19.03%).

## Discussion

In response to the global COVID-19 epidemic caused by the newly emerged coronavirus, multiple studies and clinical trials have been conducted to accelerate the finding of an effective therapeutic strategy. Passive immunization therapy using infected donors has been used previously [22-24]. Even though multiple recent studies have shown that CP does not have an impact on the prognosis of patients with severe COVID-19, there is still ongoing discussion and uncertainty surrounding the use of CP in treating severe cases of the disease [23,25]. Clinical studies show the feasibility of CP treatment for COVID-19; more studies are needed to determine the efficacy and safety of this approach according to the presence and type of comorbidities [15,26]. This study focused on understanding whether our community is ready to adapt to this therapeutic approach and whether they are willing to contribute. A total of 60.02% were male participants, most of them aged between 35 and 44 years old, while the female participants were younger.

Almost half of the participants were from the Central Saudi Arabian region, where the biggest CP clinical trials are conducted. The majority (64.61%) were graduates and 23.5% were postgraduates; this reflects that educated participants will be more interested in participating in clinical trials, knowing the value of research, and helping others. One-third (34.35%) were healthcare employees; the rest had different occupations and 41.77% had healthcare backgrounds. Interestingly, 93.53% of the participants had never previously participated in health research, as indicated in Table 1. Of the 1,051 participants, 83.54% had confirmed positive for COVID-19, 85.35% had recovered, and 82.4% had recovered and passed 14 days after their last symptoms. This indicates that they had lived the COVID-19 experience and during their illness, had looked for a cure using social media, which explains that 59.56% had heard about CP. Given that, only 4.23% had participated in CP donation and more than 50% had heard about it from social media channels, public education events could help increase awareness of the importance of participation in clinical trials [5,10].

The community shows a positive impact on participating. The great majority (83.44%) will donate CP if they are asked to join by their doctors, while 2.76% donated and participated in the CP clinical trial for COVID-19 cases before receiving this survey (see S1).

The participants responded with their motivations to participate in a CP clinical trial: 90.49% responded to saving a patient's life and 67.55% to treating a relative. This result was affected by the fact that many participants had suffered from COVID-19 and knew how bad it could be. This research highlighted the importance of clinical trials and raised awareness; 81.54% were affected emotionally by the study. Nearly all the participants (94.77%) would participate for the positive impact it would have on them, which reflects the answers from S2:A and B. The majority, 62.13%, worried about experiencing intense emotions during the research session because most had been previously infected and worried about reinfection. The motivation extends to the value of research, where 72.41% found participating in clinical research meaningful, which fit with the results in S1, while 83.44% would enroll in a clinical trial if advised by their physician. The majority agreed that clinical research is for a good cause. However, 41.29% do not know if the experience would be convenient for them, and 20.65% think it might not be. Some participants liked the research views of the research team. More than half of the participants showed negative results. Hence most participants suffered from COVID-19; 70.69% would participate knowing that they might be one of those cases. Four-fifths (80.40%) of the participants thought about the impact of national research on the patients and the country in general, and 76.02% thought it would bring value to others. This is reflected by the fact that most participants are from higher education and are affected by COVID-19, which might show a bias since they would know how it feels to have the coronavirus and the research value. Limitations were that most of the participants were in Riyadh and Western regions where the clinical trial is running. In this study, we note that the participants will participate in clinical trials if informed about them (Figure 2, Table 2).

The limitations of this study include the study design (online survey), which might lack responses from patients who do not use social media. Furthermore, only a few centers in Saudi Arabia have approved and practiced CP clinical trials. Despite these limitations, the present study conducted among patients infected with COVID-19 in Saudi Arabia shows their willingness to participate in clinical trials.

## Conclusions

The participants’ answers mainly focus on improving social media channels and emphasize education by sharing previous donor experiences. Learning from this pandemic, engaging the community in clinical trials, and educating others about the value of it by sharing experiences would promote clinical trials and encourage donations.

## Appendices

**Table 3:** Awareness level participation in a convalescent plasma clinical trial

Supplementary					
Category	Variable	N	%	Chi-square test	P- value
Have you had confirmation of COVID-19 infection with a swab test?	Yes	878	83.54	c^2^ (1,N=1051) = 472.91	p<0.001
	No	173	16.46		
Have you recovered from COVID-19?	Yes	897	85.35	c^2^ (1,N=1051) = 525.26	p<0.001
	No	154	14.65		
Has it been at least 14 days since your last symptoms?	Yes	866	82.4	c^2^ (1,N=1051) = 441.2	p<0.001
	No	185	17.6		
Before participating in this survey, had you heard about plasma donation in blood banks?	Yes	626	59.56	c^2^ (1,N=1051) = 38.441	p<0.001
	No	425	40.44		
Before participating in this survey, had you donated plasma to any blood bank in the kingdom?	Yes	45	4.28	c^2^ (1,N=1051) = 878.71	p<0.001
	No	1006	95.72		
Before participating in this survey, had you heard about the national clinical trial to treat COVID-19 critical cases with convalescent plasma from recovering COVID-19 patients?	Yes	496	47.19	c^2^ (1,N=1051) =4.230	p= 0.069
	No	555	52.81		
If yes, can you indicate the way you heard about this trial?	Friends enrolled in this study	21	4.23	c^2^ (4,N=496) = 205.77	p<0.001
	Healthcare providers	93	18.75		
	Media (video, infographics, podcast)	181	36.49		
	Other	39	7.86		
	Social media	162	32.66		
Would you enrol in this clinical trial treating COVID-19 critical cases with convalescent plasma if advised by your physician?	Yes	877	83.44	c^2^ (1,N=1051) = 470.23	p<0.001
	No	174	16.56		
Before receiving this survey, had you enrolled in the clinical trial to treat COVID-19 critical cases with convalescent plasma?	Yes	29	2.76	c^2^ (1,N=1051) = 938.2	p<0.001
	No	1022	97.24		

**Table 4:** Motivation for and psychological impact of participating in a convalescent plasma clinical trial

Category	Variable	N	%	Chi-square test	P- value
Motivation: Can you select the reasons below that led you to participate in a convalescent plasma clinical trial?					
I know that I could save a patient’s life.	Yes	951	90.49	c^2^ (2,N=1051) = 1549	p<0.001
	No	23	2.19		
	Maybe	77	7.33		
Plasma products could be used to treat my relatives.	Yes	710	67.55	c^2^ (2,N=1051) = 579.88	p<0.001
	No	103	9.80		
	Maybe	238	22.65		
The research raised emotional issues for me that I had not expected.	Yes	857	81.54	c^2^ (2,N=1051) = 1106.1	p<0.001
	No	62	5.90		
	Maybe	132	12.56		
I gained something positive from participating.	Yes	996	94.77	c^2^ (2,N=1051) = 1785.2	p<0.001
	No	21	2.00		
	Maybe	34	3.24		
I experienced intense emotions during the research session.	Yes	653	62.13	c^2^ (2,N=1051) = 416.35	p<0.001
	No	134	12.75		
	Maybe	264	25.12		
I found participating in the clinical research personally meaningful.	Yes	761	72.41	c^2^ (2,N=1051) = 770.41	p<0.001
	No	53	5.04		
	Maybe	237	22.55		
I think clinical research is for a good cause.	Yes	762	72.50	c^2^ (2,N=1051) = 757.29	p<0.001
	No	70	6.66		
	Maybe	219	20.84		
Participating in clinical research was convenient for me.	Yes	400	38.06	c^2^ (2,N=1051) = 77.768	p<0.001
	No	217	20.65		
	Maybe	434	41.29		
Psychological: Can you select from the reasons below that led you to participate in a convalescent plasma clinical trial?					
Research views from the research team.	Yes	406	38.63	c^2^ (2,N=1051) = 104.62	p<0.001
	No	196	18.65		
	Maybe	449	42.72		
I imagined I was one of these critical cases.	Yes	743	70.69	c^2^ (2,N=1051) = 665.66	p<0.001
	No	123	11.70		
	Maybe	185	17.60		
Improving public participation in national research.	Yes	845	80.40	c^2^ (2,N=1051) =1058.7	p<0.001
	No	59	5.61		
	Maybe	147	13.99		
I believe the clinical research results will be useful to others.	Yes	799	76.02	c^2^ (2,N=1051) = 893.16	p<0.001
	No	52	4.95		
	Maybe	200	19.03		
